# Understanding Users’ Engagement in a Provider-Created Mobile App for Training to Advance Hepatitis C Care: Knowledge Assessment Survey Study

**DOI:** 10.2196/52729

**Published:** 2024-11-01

**Authors:** Maximilian Wegener, Katarzyna Sims, Ralph Brooks, Lisa Nichols, Robert Sideleau, Sharen McKay, Merceditas Villanueva

**Affiliations:** 1 Department of Internal Medicine, Section of Infectious Diseases Yale School of Medicine Yale University New Haven, CT United States

**Keywords:** HIV, HCV, hepatitis C virus, interactive digital interventions (IDI), education, mobile application, user engagement, training, awareness, treatment, testing

## Abstract

**Background:**

The World Health Organization and the Centers for Disease Control and Prevention have set ambitious hepatitis C virus (HCV) elimination targets for 2030. Current estimates show that the United States is not on pace to meet elimination targets due to multiple patient, clinic, institutional, and societal level barriers that contribute to HCV testing and treatment gaps. Among these barriers are unawareness of testing and treatment needs, misinformation concerning adverse treatment reactions, need for substance use sobriety, and treatment efficacy. Strategies to improve viral hepatitis education are needed.

**Objective:**

We aim to provide a high-quality HCV educational app for patients and health care workers, particularly nonprescriber staff. The app was vetted by health care providers and designed to guide users through the HCV testing and treatment stages in a self-exploratory way to promote engagement and knowledge retention. The app is comprised of five learning modules: (1) Testing for Hep C (hepatitis C), (2) Tests for Hep C Positive Patients, (3) Treatments Available to You, (4) What to Expect During Treatment, and (5) What to Expect After Treatment.

**Methods:**

An HCV knowledge assessment survey was administered to providers and patients at the Yale School of Medicine and 11 Connecticut HIV clinics as part of a grant-funded activity. The survey findings and pilot testing feedback guided the app’s design and content development. Data on app usage from November 2019 to November 2022 were analyzed, focusing on user demographics, engagement metrics, and module usage patterns.

**Results:**

There were 561 app users; 216 (38.5%) accessed the training modules of which 151 (69.9%) used the app for up to 60 minutes. Of them, 65 (30.1%) users used it for >60 minutes with a median time spent of 5 (IQR 2-8) minutes; the median time between initial accession and last use was 39 (IQR 18-60) days. Users accessed one or more modules and followed a nonsequential pattern of use: module 1: 163 (75.4%) users; module 4: 82 (38%); module 5: 67 (31%); module 3: 49 (22.7%); module 2: 41 (19%).

**Conclusions:**

This app, created in an academic setting, is one of a few available in English and Spanish that provides content-vetted HCV education for patients and health care supportive staff. It offers the convenience of on-demand education, allowing users to access crucial information about HCV management and treatment in a self-directed fashion that acknowledges and promotes variable preferences in learning approaches. While app uptake was relatively limited, we propose that future efforts should focus on combined promotion efforts with marketing strategies experts aligned with academic experts. Incorporating ongoing user feedback and integrating personalized reminders and quizzes, will further enhance engagement, supporting the broader public health HCV elimination goals.

## Introduction

Despite the availability of highly effective, short-coursed, and well-tolerated treatment regimens, there continues to be suboptimal progress in eradicating the hepatitis C (Hep C) virus (HCV). Both the World Health Organization’s (WHO’s) Global Hepatitis Elimination Strategy and the Centers for Disease Control and Prevention’s (CDC’s) US National Hepatitis C Strategic Plan (2021-2025) have set ambitious HCV elimination targets for 2030 (curing 80% of those chronically infected) [1,2]. Published HCV care cascades in the United States have shown overall poor rates along the entire care cascade. Before the introduction of direct-acting antivirals (DAAs), a meta-analysis by Yehia et al [3] showed that among those with HCV mono-infection, only 50% were diagnosed and aware of their infection, 43% had access to outpatient care, 27% had HCV RNA confirmed, 16% were prescribed treatment, and 9% achieved sustained virologic response after 12 weeks of treatment [3]. In the current DAA era, HCV viral clearance cascades continue to show significant gaps in viral testing and cure or clearance rates. Further, 1 study using private laboratory data in the United States estimated that 12% of persons in the database with evidence of HCV infection did not receive follow-up viral testing, and only 34% of those chronically infected achieved viral cure or clearance [4]. A similar state-specific study found a median cure or clearance rate of 34.5% (range 10%-51%) with variability by state [5]. These results indicate that while some states have significant testing gaps, all states have substantial gaps in cure or clearance rates [5]. Many barriers exist on patient, clinic, institutional, and societal levels that contribute to HCV testing and treatment gaps [6]. On patient and clinic levels, there are multiple coexisting barriers such as lack of understandable information (including misinformation) concerning the need for testing, liver health, the efficacy of available treatments, adverse treatment reactions, and the impact of substance use all of which could benefit from enhanced approaches to health literacy [7,8]. Strategies to improve viral hepatitis education are needed to achieve the national viral hepatitis strategic goals.

Well-resourced and content-vetted educational trainings have typically focused on clinical providers, particularly prescribers. Examples include online trainings from the national AIDS Education and Training Center and the University of Washington [9,10]. Project ECHO launched by the University of New Mexico focuses on case-based sessions delivered virtually [11]. Frequently updated guidelines are available from the AASLD (American Association for the Study of Liver Diseases) and the Infectious Disease Society of America and are highly technical and targeted at state-of-the-art clinical care [12]. Approaches toward patient education are more variable given the proliferation of internet-based content that is often not curated by medical professionals. The traditional standard has been for providers to educate patients primarily during face-to-face visits, but a common barrier to HCV treatment is patient loss to follow-up by clinics [13]. To overcome this, 1 approach has been to use digital interventions, including smartphone apps. Further, 2 systematic reviews focusing on digital interventions promoting sexual health found they increased knowledge across medical topics and are as effective, if not more, as in-person minimal intervention education [14,15]. Additionally, the studies revealed that digital interventions positively impacted patient behavior and attitudes toward safer sex practices as well as with HIV testing, prevention, and treatment, potentially improving clinic attendance and treatment adherence [14,15]. The convenience of digital materials, allowing users to access content privately, is particularly valuable for sensitive topics [14].

Hep C–focused educational apps are relatively limited with only a dozen high-quality apps available on the Google Play Store or Apple App Store [16]. Moreover, most of these apps (67%) are targeted toward health care providers, particularly prescribers, with only 2 apps intended for patients [16] with scant data evaluating their effectiveness. Existing literature available for HCV education is focused on the comanagement of opioid use disorders and do not provide targeted information on general HCV treatment and cure [17-19]. In response to these gaps as well as needs assessment and feedback from multidisciplinary stakeholders, we developed an app to address the lack of HCV educational resources available to patients and health care supportive staff. The app guides the user through the HCV treatment stages in a self-exploratory way. It is designed to replicate the patient experience during HCV testing and treatment and serves as a companion to medical care [20]. In this report, we aim to evaluate the initial roll-out of this HCV educational app designed for patients and health care workers and speculate on future implementation strategies.

## Methods

### Study Design

This activity was funded by the Health Resources and Services Administration (HRSA) Special Projects of National Significance initiative entitled *Jurisdictional Approach to Curing Hepatitis C among People of Color Living with HIV*. The Yale School of Medicine (YSM) was funded to work with the Connecticut Department of Public Health and 11 Connecticut-based HIV clinics. Our project was called the *Connecticut Quantification Evaluation and Response: HIV/HCV Elimination in Persons of Color*. HRSA contracted with RAND Corporation to serve as the project evaluator.

### Needs Assessment

A knowledge assessment survey created by RAND was administered at baseline to clinic health care workers (prescriber and nonprescriber) and patients to identify knowledge gaps that were thought to be barriers to promoting entry into HCV treatment. Further, 2 different surveys were administered (33 questions for health care workers and 41 for patients). Patient questions included the following categories: HCV screening and testing, HCV treatment and medication, substance use and mental health experiences, HCV knowledge and beliefs, and demographic and socioeconomic information. Clinic health care worker questions included: HCV/HIV coinfection knowledge and beliefs, HCV and HIV treatment and medication protocols, demographic and practice information, and patient care services available. Both surveys were used to define specific areas to target educational programs. Among health care worker gaps (unpublished data), disparities in knowledge about DAA treatments between physicians and nonphysicians were seen; knowledge gaps concerning HCV treatment for patients with co-occurring substance use and mental health issues were noted in all health care workers. Patient knowledge gaps included a lack of knowledge about HCV treatment, even among those who had previously received it, as well as misunderstandings about HCV transmission and symptoms. There were 75 clinic health workers (33 physicians, 7 advanced practice registered nurses or physician assistants, 35 nonprescribers [eg, registered nurses, case managers, outreach workers, etc]), and 220 patients who participated. Given the specific knowledge gaps identified, the YSM team decided to design a new grant-funded mobile app.

We used the components of Hep C management simplification outlined by Kapadia and Marks [20] as a starting point for app design. A group of clinicians and staff from YSM and the 11 participating clinics met between September 2018 and May 2019. Feedback from this group was solicited during scheduled programmatic team meetings and was incorporated into the app design process. There were three goals identified for the app: (1) inform patients and health care workers about HCV progression and facilitate conversations with providers and other members of the health care team, (2) educate patients and health care workers about the importance of HCV screening and treatment, and (3) assist in improving knowledge dissemination of HCV disease and its management.

### Development Process

The original guiding principle for the app was to build it primarily from a patient perspective with a focus on patient-centered concerns and included (1) mirroring patients’ experience during and after treatment; (2) content aimed at no higher than a middle school educational level; (3) use of on-demand learning, with the ability to skip between and within topics or repeat sections as needed; (4) incorporation of content scaffolding to promote interactive learning: questions-answer-application-feedback; (5) available on both the Android and iOS mobile platforms, with alternative portal access via the web for those lacking mobile device access; (6) Spanish and English versions; and (7) free to download or install (beyond the cost of data).

The HEP C (hepatitis C) Training app was designed by members of the YSM study team, with content generated based on input from multi-stakeholders involved in the grant. Further, 2 Connecticut-based third-party developers were used: Love Local Design provided the graphic design and process flow visualization; Amston Studio provided the app coding, data structures, servers, and managed the technical part of the release. The mobile app was created in React Native (Meta Platforms, Inc) and uses a SaaS backend API service via a token-based protocol. The backend service is hosted in Amazon Web Services using EC2, S3, and RDS for the MySQL database. The app also includes a language toggle allowing users to select Spanish as their preferred language. After the initial design, the app was piloted among clinic health care workers (physician and nonphysician) from participating clinics, community health workers, and selected patients for ease of use and patient accessibility. Feedback was incorporated into the final design.

### App Design and Features

**Figure 1:**
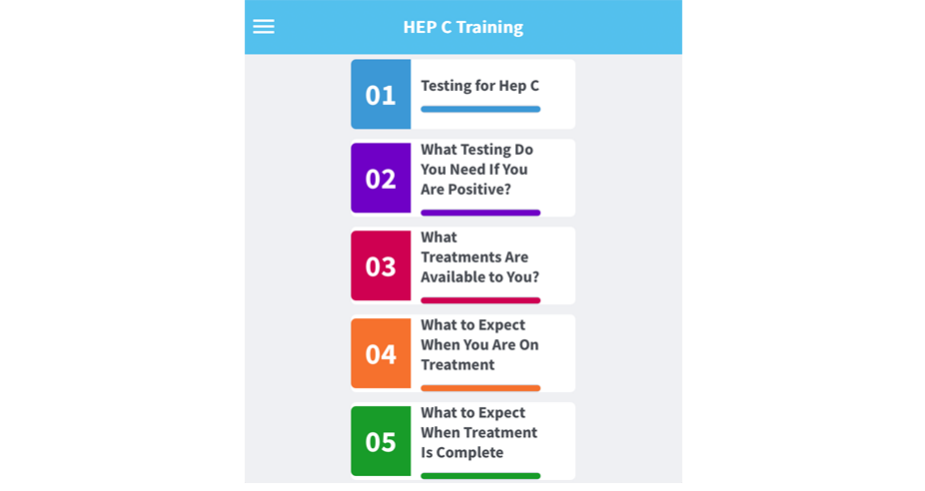
Home page of the HCV Training app showing available training modules.

The app is organized into 5 modules based on the primary care provider framework for management simplification of HCV as shown in Figure 1 [20].

**Figure 2:**
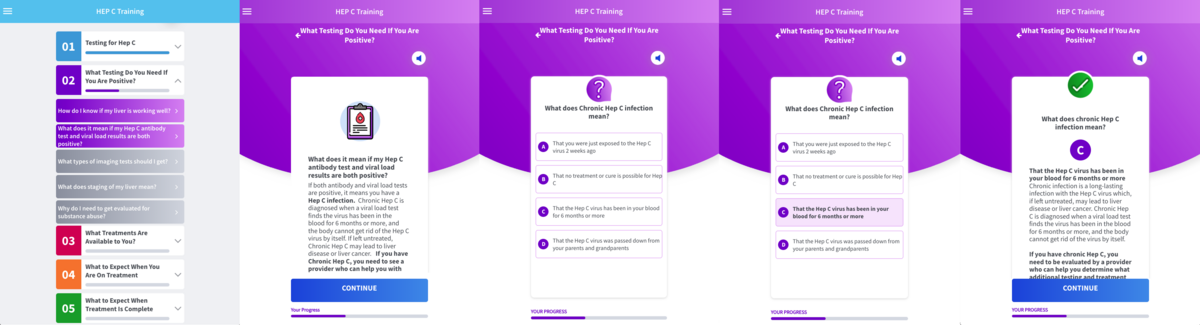
Flow within module 2 (What Testing Do You Need If You Are Positive) showing content, question, answer, and then feedback.

Module 1: Testing for Hep C, module 2: What Testing Do You Need If You Are Positive, module 3: What Treatments Are Available to You, module 4: What to Expect When You Are On Treatment, and module 5: What to Expect When Treatment Is Complete. Each module can be accessed on the home page of the app and is color-coded and expandable for user-friendly navigation. Although the sections are intended to be used in numeric sequence, users may review them in any nonsequential order that interests them. Each module is followed by formative assessments, including 29 quiz questions across the modules to solidify user knowledge acquisition. An example of the nature of the app is shown in Figure 2.

Use of the app is anonymous; names and personal information are not recorded; however, each device is assigned a device ID upon activation and data such as correctly submitted quiz responses, length of time spent per section, and rate of topic visits, are recorded by the servers using this ID. Users can track their progress and return to previous sections at any time. There are additional sections including a resources dropdown page (accessed through the home page) and pages for Helpful Links and a Glossary. The Helpful Links section contains links to external websites such as the CDC’s HCV and patient education resources, National Institutes of Health HCV frequently asked questions, CDC’s Patient Education Resources, HCV info on the American Liver Foundation website, and the Mayo Clinic’s website. The Glossary permits quick checking of the terminology often used during medical appointments that users might not be familiar with. An About section is also available on the app home page and describes the primary goal of the app. The Language page allows switching between English and Spanish. Additional functionality includes a text-to-speech subroutine provided in Spanish or English, as well as a web version of the application, created in React JavaScript for use on computers. The web version does not require any download or installation privileges on the host device and is ideal for presenting and using the app in large-group training sessions, seminars, conferences, or for those who might seek to use it in a setting where the target equipment has multiple users (eg, public library). Additional materials such as a user guide are available on the website in Spanish and English and assist users with app installation.

### App Dissemination

Once finalized, the app was put into production in November 2019 and released on the Apple App Store and Google Play Store, and available for use on a computer via a web browser. The app was available to the general public, but grant-related dissemination by the YSM team was largely focused on persons with HCV or those who work in the HCV field, primarily health care nonprescriber staff (eg, case managers, outreach workers, etc). Advertising the app was carried out on study websites [21], collaborative meetings with study partners and stakeholders, at conferences, seminars, and similar events, as well as by word-of-mouth.

### Data Analysis

Analysis was conducted for datasets supplied by Amston Studios exported directly from the server in November 2022 as Excel (Microsoft Corp)-formatted spreadsheets. The datasets provided retrospective data on app use for 3 years. An anonymous unique device ID was used to distinguish between individual devices (eg, users), and allowed for monitoring progress as modules were completed. The time duration of the app was calculated using the app installation date or time and the most recent app activation date or time. Descriptive analysis was also conducted for user device type (phone or web-based) to calculate the percentage of each along with an analysis showing device type within each time with application category. The modules completed by unique user data elements were determined by cross-referencing module names with device ID and tallying to obtain the number of modules completed. A descriptive analysis was conducted to see module completion percentages for the 5 available modules.

### Ethical Considerations

This study was approved by the Yale Institutional Review Board (protocol #2000025960). Informed consent was waived as all survey and app data were collected anonymously. Clients who participated in the knowledge assessment survey were compensated with a US $20 gift card upon completing the survey. All data were deidentified to ensure participant privacy and confidentiality.

## Results

### Accession Characteristics

The app was accessible via web or mobile device (downloads). From November 2019 to November 2022, there were 561 app accessions from 29 different countries with the United States having 84.5% (n=474). Among these, there were 216 distinct users (based on device IP address) who further accessed the training modules from 16 countries; 188 (87%) were in the United States. To ensure an end user experience that remained as anonymous and confidential as possible, no demographic data were collected for users who downloaded the app, so none is available for analysis.

### Time Use of Accessions

Table 1 shows a breakdown of interface types (web vs mobile device) and time having the app available for the 561 app accessions through November 2022. The time with the app represents the amount of time between when a user first downloaded the app to when it was most recently opened. Mobile use accounted for 63.4% (n=356) of the app users and 36.6% (n=205) used web-based interfaces. Overall, 216 (38.5%) used the app for any amount of time beyond installation (>0 min). Of the 216, a total of 151 (69.9%) used the app for up to 60 minutes and 65 (30.1%) used it for 1 day or more. Additionally, for users who accessed the app for up to 60 minutes (N=151), the median time spent with the app was 5 (IQR 2-8) minutes. For users who accessed the app for 1 day or more after downloading (n=65), the mean time between initial download and last use was 44 (SD 33, median 39, IQR 18-60) days. After excluding users with no time spent on the app, mobile users accounted for 81.5% and web users 18.5% (N=216).

**Table 1:** Time use of the app by the number of downloads (November 2019 to November 2022; N=561 accessions).

Time on app	Mobile (n=356), n (%)	Web (n=205), n (%)	Total, %
0 min	180 (32.1)	165 (29.4)	61.5
>0 to 30 min	117 (20.9)	20 (3.6)	24.5
30 to 60 min	11 (1.9)	3 (0.5)	2.4
1 to 7 d	10 (1.8)	8 (1.4)	3.2
8 to 14 d	5 (0.9)	1 (0.2)	1.1
15 to 30 d	4 (0.7)	1 (0.2)	0.9
31 to 60 d	10 (1.8)	2 (0.4)	2.2
61 to 90 d	4 (0.7)	0 (0)	0.7
91 to 120 d	3 (0.5)	0 (0)	0.5
121 to 150 d	5 (0.9)	0 (0)	0.9
>150 d	7 (1.2)	5 (0.9)	2.1
Total	356 (63.4)	205 (36.6)	100

### Analysis of Patterns of Module Use

**Figure 3:**
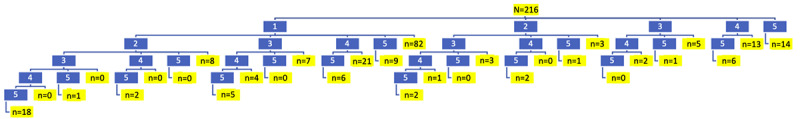
Sequence of modules accessed by number of users who accessed modules (N=216).

Given that the app design reflected the clinical sequence from testing to treatment and posttreatment, we were interested in seeing the temporal sequence of how users navigated the modules. Further, 216 users accessed the training modules of the app at least once. Figure 3 shows how these app users followed an incomplete, inconsistent, and nonsequential pattern of module use, with various modules being skipped or users not starting from module 1. To streamline this diagram and analysis, possible pathways were consolidated by ignoring the order of actual module completion and considering only what content (eg, modules) were accessed, with the modules being listed serially from 1 to 5. The numbers highlighted in blue represent the module numbers and the branches connecting them indicate which modules were used. The numbers highlighted in yellow represent the number of users in a particular content pathway. The pathway branching is based on whether a module was accessed by the user; with the lowest modules in the series considered first. For example, in the first line, the yellow highlighted number 14 indicates the number of users who went straight to module 5 (posttreatment) and only completed that module. Similarly, in the second line, the yellow highlighted number 82 represents the number of users that only completed module 1. On the same line, there were 3 that completed the second module only, 5 that completed the third module only, and 13 that completed the fourth module only. On the third line on the left part of the diagram, the yellow highlighted number 8 indicates the number of users who completed modules 1 and 2. Below that, the number 2, highlighted in yellow, indicates the number of users that completed modules 1, 2, 4, and 5.

The module with the highest engagement was module 1 (Testing for Hep C), accessed by 163 (75.4%) users. Module 4 (What to Expect when You Are on Treatment), was the next highest with 82 (37.9%). Next, there were 67 (31%) users who accessed module 5 (What to Expect when Treatment is Complete), then 49 (22.6%) who accessed module 3 (What Treatments are Available to You?), and 41 (19%) who accessed module 2 (What testing Do You Need if You Are Positive?).

### Use of Quiz Questions Within Modules

The design of this mobile app was reflected in a series of questions to test app users’ comprehension of the content. There are 27 questions total, and users can skip and answer any question multiple times. Table 2 shows the percent of correct answers for each question and is based on distinct user IDs and user answers. Correct answer percentages ranged between 53.4% and 92% with a median score of 78.8% (IQR 65.2%-85.2%). Questions that scored lowest in each module (<70% correct) included: “what is Hep C?” (n=67, 63.2%), “how does Hep C affect my liver and my health?” (n=49, 59%), “how is the Hep C test done?” (n=55, 53.4%), and “what do my test results mean?” (n=43, 65.2%) for module 1; how do I know if my liver is working well? (n=31, 66%), what does staging of my liver mean? (n=22, 62.9%), and why do I need to get evaluated for substance abuse? (n=24, 55.8%) for module 2; and how should I make sure I don’t miss any pills? (n=17, 65.4%) for module 4. Modules 3 and 5 did not have any answers below 70%.

**Table 2:** Percentage of current quiz question answers per module.

Module number quiz question, and number of correct answers percent correct		Correct, n (%)
**1: Testing for** **Hep C^a^**		
	What is Hep C? (N=106)	67 (63.2)
	How does Hep C affect my liver and my health? (N=83)	49 (59)
	How did I get Hep C? (N=61)	53 (86.9)
	How do I know if I have Hep C? (N=63)	50 (79.4)
	How is the Hep C test done? (N=103)	55 (53.4)
	What do my test results mean? (N=66)	433 (65.2)
	If my antibody test result is negative, what can I do to keep my liver healthy? (N=45)	38 (84.4)
**2: What testing do you need if you are positive?**		
	How do I know if my liver is working well? (N=47)	31 (66)
	What does it mean if my Hep C antibody test and viral load results are both positive? (N=37)	30 (81.1)
	What types of imaging tests should I get? (N=30)	22 (73.3)
	What does staging of my liver mean? (N=35)	22 (62.9)
	Why do I need to get evaluated for substance abuse? (N=43)	24 (55.8)
**3: What treatments are available to you?**		
	How will my provider decide which Hep C treatments are right for me? (N=45)	38 (84.4)
	What medications are available to treat Hep C and how successful are the treatments? (N=35)	30 (85.7)
	Which pharmacy will I work with? (N=33)	26 (78.8)
	Will insurance cover my treatment? (N=27)	23 (85.2)
	If I am using drugs, does this mean I can’t get treatment for Hep C? (N=29)	23 (79.3)
**4: What to expect when you are on treatment?**		
	What are the side effects of treatment? (N=24)	19 (79.2)
	How should I make sure I don’t miss any pills? (N=26)	17 (65.4)
	How do I know if my liver is working well? (N=19)	17 (89.5)
	What kinds of appointments and lab tests should I expect during treatment? (N=22)	19 (86.4)
	How do I manage my substance use issues during treatment? (N=22)	19 (86.4)
**5: What to expect when treatment is complete?**		
	What does it mean to be cured? (N=30)	21 (70)
	What other lab tests will I need to get? (N=30)	21 (70)
	Will I need additional imaging tests? (N=21)	18 (85.7)
	Will I need to see a liver specialist? What for? (N=29)	24 (82.8)
	What should I do to make sure my liver is healthy? (N=50)	46 (92)

^a^Hep C: hepatitis C.

## Discussion

### Principal Findings

This study describes our approach to developing and implementing an HCV educational mobile app designed to improve critical educational gaps that represent barriers to improving the HCV testing and treatment cascade. The advantages of our app include development by subject matter experts, testing, and incorporating feedback from end users to ensure an appropriate educational level, and availability in Spanish. We analyzed 3 years of data resulting in important insights into user behaviors and found suboptimal engagement at levels of initial download and subsequent progress through the learning modules. We discuss potential lessons learned to improve future implementation.

The WHO defines mobile health (mHealth) as “the medical and public health practice supported by mobile devices such as mobile phones.” The National Institutes of Health defines mHealth as the “use of mobile and wireless devices to improve health outcomes, healthcare services and health research.” There are multiple potential advantages of mHealth including assistance in chronic disease management and promoting general wellness motivation. Ideally, such platforms can promote patient engagement, that is, enhancing patients’ ability to fully participate in health care, helping them to be “equipped, enabled and empowered” regarding their health [22]. Use of such platforms can occur through generalized e-learning (self-learning or on demand) or by promotion of health parameter tracking through available monitoring or tracking features. There are many potential advantages to mHealth approaches for patients such as easy installation, constant availability, ease of use with rapid update capacity, and customizability. Systemically, these advantages can translate to broader dissemination and scalability, and the ability to outsource educational efforts with the theoretical capacity to be a low-resource solution to help patients engage in health care. For e-learning, the advantages include the accommodation of individualized learner needs, the ability to be self-paced, and the rapid delivery of content with consistent quality.

Despite these potential advantages, there is surprisingly little data to support the effectiveness of these mobile apps for outcomes such as improving patient care [23,24]. Studies looking at user engagement with mobile apps in the mental health field have pointed out that no standard metrics exist to measure app engagement; these authors reviewed user engagement with an mHealth smartphone app designed to promote education and tracking of mental health issues with potential access to a mHealth provider [24]. They found that 57.9% of eligible users never downloaded the app for their study, 14% used the app 1 day after downloading, and 74% of users who downloaded the app stopped engaging after 10 uses [24]. This pattern of app use is similar to our findings. These researchers used a Delphi process to evaluate potential reasons for low smartphone app engagement and uncovered the following themes: (1) poor usability: apps were not designed with servicing the user in mind such as technically challenging to use, needing user time to enter data, and lacking of ability to sustain user interest; (2) lack of user-centric design: they do not solve problems users cared about, lack emotional support, track symptoms not universally valued, and are not helpful in emergencies; (3) privacy concerns: health data often exist outside of health care regulation, concern that the app developer may market data, and uncertainty regarding interpreting app privacy; and (4) trust concerns: concern that information was not developed using evidence-based information.

These authors made several recommendations to improve user engagement such as involving end users in design and testing, ensuring clinicians are part of the design process, ensuring a system for quality control such as endorsement by professional societies, and embedding the app into diverse systems and settings where users can engage beyond the clinical setting [24]. They concluded that more research is needed to better characterize best practices for the design, testing, and implementation of mobile apps [24]. Many of these recommendations were taken into consideration in the design of our HEP C app.

Few studies are looking at the design and implementation of mobile apps, specifically to improve HCV care. Tofighi et al [25] assessed technology preferences to enhance HIV and HCV care among patients with substance use disorders and found patients preferred technological (phone or web-based) interventions over in-clinic or in-person visits for receiving HIV- and HCV-related information [25]. Hochstatter et al [17] conducted a randomized controlled trial among patients at an opioid use disorder clinic using an mHealth intervention aimed at relapse prevention and improving HCV care continuum outcomes. The researchers found a trend in increased HCV testing, particularly for persons who share injection equipment, suggesting the intervention may improve HCV testing rates especially for those with the highest risk of infection [17]. Rodrigues et al [16] reviewed publicly available mobile apps designed to support HCV treatment using a Mobile Application Rating Scale, a 23-item scale suggested to assess app quality [16]. Among 316 potentially available applications, only 12 met the objective Mobile Application Rating Scale criteria for engagement, functionality, aesthetics, and information quality. Of the 12 apps, designers included commercial developers (n=7), individual developers (n=2), university-designed (n=1), nongovernmental (n=1), and governmental organizations (n=1). The target audience for these were primarily health care providers (n=8, 67%) followed by patients (n=2, 17%), both (n=1, 8%), and unknown (n=1, 8%) [16]. Studies of the implementation of e-learning approaches in HCV are also limited. True app use studies are lacking. In a more traditional approach, Ochalek et al [18] used a clinic-based single-visit educational modality among adults with opiate use disorder in a clinic-based program that assessed HIV and HCV knowledge at baseline, and after the roll-out of an iPad-based flipbook and video they found sustained and significant improvement in HIV and HCV knowledge [18].

In a more deliberate design to promote interaction within the app, Garg et al [26] looked at a mobile app for self-learning on HIV prevention in Indonesia using a user-centric design with features including private messaging to providers, games section, and peer-facilitated administration using youth peer leaders [26]. They found app use led to improvements in HIV-related knowledge, HIV testing, and behavioral outcomes related to safer sex and drug use practices.

Detailed data on patterns of app usage is limited. In our study, we observed the nonordinal progression through the app, meaning users did not go from module 1 to module 5 in order. As expected, the module accessed most often was module 1 (n=163, 75%), as it is the first module listed once the app is open. The next 2 modules accessed most were 4 (n=82, 38%) and 5 (n=67, 31%). This key finding shows which topics users are most interested in completing and interestingly, these topics correspond with the testing and treatment gaps found along the HCV clearance cascade [27].

The biggest challenge was eliciting user engagement in the app. While 88.4% (N=561) of the total users spent 0-60 minutes with the app, 61.5% (N=561) downloaded but never used it. Lack of user engagement has been a well-documented issue associated with various app types [16,28,29]. A systematic review of HCV-specific learning apps showed user engagement as the lowest-scoring measure for all apps observed [16]. Addressing this gap and increasing patient engagement has been shown to enhance knowledge and improve health literacy and motivation [30]. It is important to understand what drives user engagement to increase app use frequency [31]. Gamification or financial incentives may be more effective approaches to increase engagement as has been seen in some medication adherence mobile apps [32]. Moreover, marketing of mobile apps used for patient health literacy may be more effective if targeted to providers and organizations for use in the context of a health literate care model where learning is overseen and supported during clinical encounters [33].

Data from the app provides insights into topics users presumably find most interesting or relevant which can be useful for developing future HCV training opportunities, either online or in person. Discovering which questions users scored lowest on can also help enhance future trainings by identifying subjects that need more focus. Incorrect responses to questions were notably related to liver health and HCV testing, suggesting that the clinical provider content creators may have overestimated health literacy in these domains.

Dissemination of the mobile app showed initial uptake but then fell off. We speculate that using this as a stand-alone approach, particularly for patients, may have its limits. Several strategies for engagement have highlighted the need for contact and reinforcement with providers which was not built into this approach given labor intensiveness and lack of capacity [26,34]. We initially tried to create a hybrid approach where patients would do a group training using the app in concert with a substance use counselor or other trainer; we found that such a group activity was highly appealing and engaging to patients who answered questions with input from each other and the trainer. We had planned to augment such group activities to guide the use of the app, however, the COVID-19 pandemic stopped this approach. Similarly, the institution of COVID-19 restrictions on in-person activities ended typical advertising and marketing methods that would have occurred in clinics and substance use treatment programs during regular patient or client visits with care providers. We speculate that the use of this app as part of a recommendation by a clinical provider, to be administered in individual or group settings, with available feedback would be an effective implementation strategy as individual self-directed learning may have been affected by user fatigue and need for further expert guidance. Clinic staff may also benefit from incorporating the use of the app as part of staff in-service requirements.

### Limitations

The COVID-19 pandemic began during app dissemination, greatly reducing our dissemination activities. We did not collect demographic data on our users and are unable to determine, for example, which users are patients, providers, or other members of the public. Additionally, satisfaction survey data were not collected, preventing us from ascertaining why users chose the modules they did. Furthermore, due to the structure of the app’s data collection, we were unable to determine the sequence in which users completed their modules. We identified unique app users by their device IP addresses, but we could not distinguish users who accessed the app from multiple devices. Although we believe this situation is rare, it represents a limitation, as some users may have been counted more than once if they accessed the app from more than one device. Finally, we did not have a dedicated patient focus group to provide more robust input into assessing usability; however, we did have limited patient feedback during the pilot testing phase.

### Conclusion

The WHO and CDC have set ambitious goals for HCV elimination, but a lack of patient and health care worker education may be a limiting factor. Traditionally, patient education has occurred during face-to-face clinic visits, so gaps in education will persist for patients who miss appointments and have limited time due to competing health needs. We developed an app that provides well-resourced and content-vetted HCV education for patients which can be accessed anytime and anywhere. The app’s main utility is offering on-demand education, enabling patients to access crucial HCV management and treatment information at their own pace, potentially enhancing their understanding and engagement with their care. Future efforts should target marketing strategies that complement the app’s strengths, driving visibility and uptake. Additionally, incorporating app use within clinic visits for patients and clinic support staff could enhance education and overall engagement and promote HCV elimination goals.

## Abbreviations

AASLD: American Association for the Study of Liver Diseases
CDC: Centers for Disease Control and Prevention
DAA: direct-acting antiviral
HCV: hepatitis C virus
Hep C: hepatitis C
HRSA: Health Resources and Services Administration
mHealth: mobile health
WHO: World Health Organization
YSM: Yale School of Medicine
